# A novel approach to quantifying the spatiotemporal behavior of instrumented grey seals used to sample the environment

**DOI:** 10.1186/s40462-015-0047-4

**Published:** 2015-07-08

**Authors:** Laurie L Baker, Joanna E Mills Flemming, Ian D Jonsen, Damian C Lidgard, Sara J Iverson, W Don Bowen

**Affiliations:** Department of Biology, Dalhousie University, Halifax, B3H 4R2 Canada; Department of Mathematics and Statistics, Dalhousie University, Halifax, B3H 4R2 Canada; Department of Biological Sciences, Macquarie University, North Ryde, Sydney, NSW 2109 Australia; Population Ecology Division, Bedford Institute of Oceanography, Department of Fisheries and Oceans, Dartmouth, B2Y 4A2 Canada

**Keywords:** Animal movement, Bioprobe, Marine acoustics, Ships of opportunity, Ocean tracking network

## Abstract

**Background:**

Paired with satellite location telemetry, animal-borne instruments can collect spatiotemporal data describing the animal’s movement and environment at a scale relevant to its behavior.

Ecologists have developed methods for identifying the area(s) used by an animal (e.g., home range) and those used most intensely (utilization distribution) based on location data. However, few have extended these models beyond their traditional roles as descriptive 2D summaries of point data. Here we demonstrate how the home range method, T-LoCoH, can be expanded to quantify collective sampling coverage by multiple instrumented animals using grey seals (*Halichoerus grypus*) equipped with GPS tags and acoustic transceivers on the Scotian Shelf (Atlantic Canada) as a case study. At the individual level, we illustrate how time and space-use metrics quantifying individual sampling coverage may be used to determine the rate of acoustic transmissions received.

**Results:**

Grey seals collectively sampled an area of 11,308 km ^2^ and intensely sampled an area of 31 km ^2^ from June-December. The largest area sampled was in July (2094.56 km ^2^) and the smallest area sampled occurred in August (1259.80 km ^2^), with changes in sampling coverage observed through time.

**Conclusions:**

T-LoCoH provides an effective means to quantify changes in collective sampling effort by multiple instrumented animals and to compare these changes across time. We also illustrate how time and space-use metrics of individual instrumented seal movement calculated using T-LoCoH can be used to account for differences in the amount of time a bioprobe (biological sampling platform) spends in an area.

## Background

The miniaturization of environmental sensors and acoustic tags has allowed these instruments to be deployed on an increasing number of animals [1–4]. Paired with GPS satellite location telemetry, animal-borne instruments such as temperature-salinity (CTD) tags, underwater cameras, and acoustic transceivers allow for the collection of spatially-linked, fine-scale information about the host and the marine environment at a scale relevant to the animal’s behavior [1, 5].

Bioprobes, individual animals equipped with sampling instruments (e.g., CTD tags, acoustic transceivers), are not constrained by the same financial and logistic constraints as human sampling platforms and fixed acoustic-receiver arrays. They therefore have the potential to advance our understanding of the physical environment and species interactions in habitats that are inaccessible and/or inhospitable to humans [6]. However, this method of sampling the physical or biological environment differs markedly from traditional vessel-based surveys. We define sampling as the collection of any type of data about the physical or biological environment (e.g., temperature, acoustic noise). The data a bioprobe collects are intrinsically linked to the bioprobe’s behavior. Consequently, sampling locations are non-random in space and time, and we are unable to predetermine where a particular instrumented animal may go, although we may know general patterns in their movement. For example, certain marine mammals tend to forage more intensively at given points during their seasonal cycle. When sampling is conducted simultaneously by multiple instrumented animals carrying identical sensors, the overall coverage and intensity of sampling is a result of the total sum of their movements. In these cases, sampling effort can be viewed in terms of collective time spent in an area. In order to interpret the physical and biological data collected using animal-borne instruments, it is necessary to quantify sampling intensity and coverage by individual and multiple instrumented animals.

Ecologists have developed a suite of methods for identifying the area(s) used by an animal (e.g., home range) and those used most intensely (e.g., utilization distribution) based on location data (see [7] for a review of these methods). However, few have extended these models beyond their traditional roles as descriptive 2D summaries of point data, or employed these methods to study the sampling effort of instrumented animals. Here we use a specific type of home range method, Local Convex Hull (LoCoH), which estimates an animal’s utilization distribution based on local nearest-neighbour minimum convex polygons (MCP). MCPs are constructed from the relative frequency distribution of animal locations, using density as a third dimension to portray the intensity of area use [8, 9]. LoCoH methods have been shown to outperform traditional kernel-smoothing techniques in excluding areas known not to be used [9]. They are therefore appropriate for areas that incorporate distinct habitat, geographical, or physical boundaries [9]. These attributes make LoCoH methods particularly well suited to study collective area use of multiple organisms that exhibit possibly diverse individual space-use patterns. Recent developments have expanded these methods to include time in the construction and aggregation of MCPs: T-LoCoH [10]. T-LoCoH offers an advantage over traditional approaches because it further improves the user’s ability to partition area use and study patterns through time [10]. The concept of a utilization distribution can be extended to describe bioprobe sampling effort by calculating total area sampled and intensely sampled area.

We demonstrate how the home-range package, T-LoCoH [10], can be extended to characterize and quantify collective sampling effort of multiple bioprobes across time using grey seals (*Halichoerus grypus*) equipped with Global Positioning System (GPS) tags and acoustic transceivers as a case study. Acoustic transceivers are able to record coded acoustic transmissions that can be uniquely identified based on the intervals between a series of acoustic pings. Instrumented with GPS tags and acoustic transceivers, grey seals effectively become geo-referenced mobile acoustic receiving stations with the ability to continuously record coded acoustic transmissions emitted by transmitters deployed on fish and other grey seals.

Recorded transmissions present a unique opportunity to study the spatial and temporal patterns of associations between grey seals and potential prey species instrumented with acoustic tags such as Atlantic cod (*Gadus morhua*) and salmon (*Salmo salar*) [11]. In addition, because many fish do not surface, precluding the use of GPS tags, detections of instrumented fish recorded by bioprobes can also provide valuable information about the location of these fish. Grey seals are large, size-dimorphic, marine carnivores that exhibit marked seasonal changes in distribution, diet, and foraging effort according to seasonal changes in their energy needs and changes in the availability of their prey [12–17]. In order to understand the spatial and temporal nature of associations, it is necessary to account for the time spent by individual seals in certain areas. In a second application of the T-LoCoH package, we illustrate how time and space-use metrics derived using T-LoCoH can be used to account for differences in the amount of time an individual bioprobe spends in an area.

## Results and discussion

**Fig. 1:**
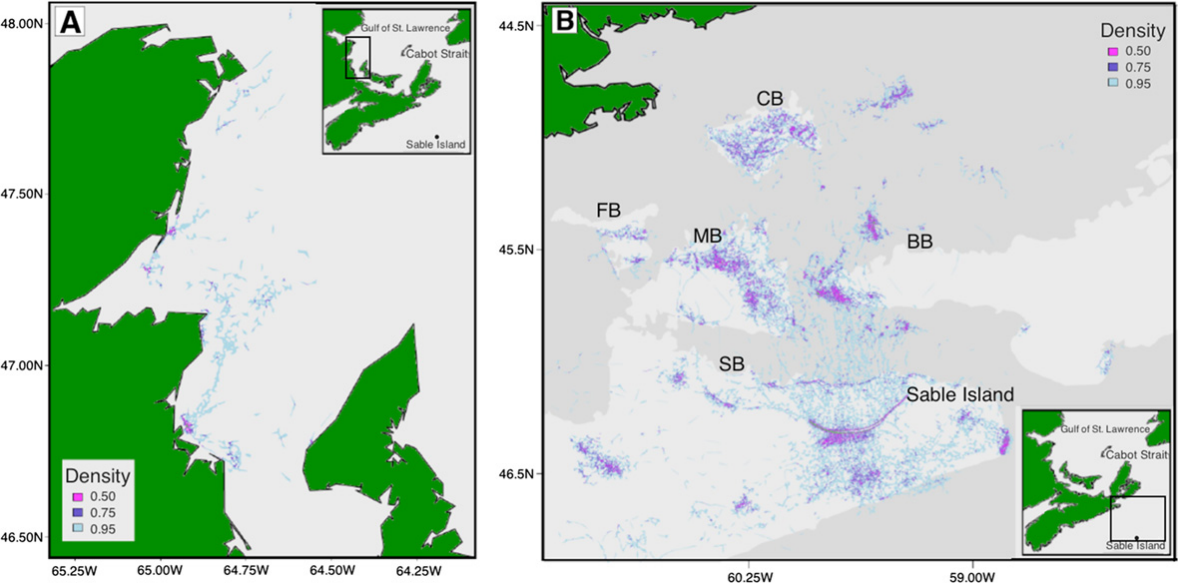
Collective area used by all bioprobes. Grey seal bioprobes collectively used an area of 11,308.28 km ^2^ (light blue, 95 % density quantile) and intensely used an area of 31.07 km ^2^ (purple, 25 % density quantile). One instrumented seal travelled to the southern Gulf of St. Lawrence and was used to study individual area use (**a**). The majority of instrumented seals stayed on the Scotian Shelf surrounding Sable Island and were used to study collective area use (**b**)

In 2011, grey seals (n =16) collectively used an area of 11,308 km ^2^ (95 % density quantile), and intensely used an area of 31 km ^2^ (25 %) during the 7-month, post-moult and pre-breeding period (June-December) (Fig. 1).

### Individual area use

In September, 40 transmissions were received, of which more than half (52.5 %) were received at the most intensely used part of the seal’s area use (25 % density quantile). Increasingly fewer transmissions were received at the 50 %, 75 %, and 95 % density quantiles with 8, 4, and 2 transmissions received, respectively (Table 1).

**Table 1:** Time-and space-use metrics for polygons associated with receipt of acoustic transmissions. The metrics are summarized by density quantile. These density quantiles represent intensity of sampling coverage (25 % = high intensity, 75 % = low intensity). Polygon area and occupancy time are used to calculate the overall sampling effort (km ^2^/h) and estimate the transmission reception per unit sampling effort (TPUE)

Density quantile	Number of transmissions	Mean area (km ^2^)	Mean time (h)	TPUE (km ^2^/h)
0.25	21	0.21 ± 0.10	1.30 ± 0.16	0.74 ± 8.94
0.5	8	2.51 ± 1.18	2.58 ± 0.78	0.62 ± 1.83
0.75	4	6.59 ±2.87	4.17 ± 1.87	0.03 ± 1.26

N.B. Seven transmissions were received outside of the 75% density quantile

**Fig. 2:**
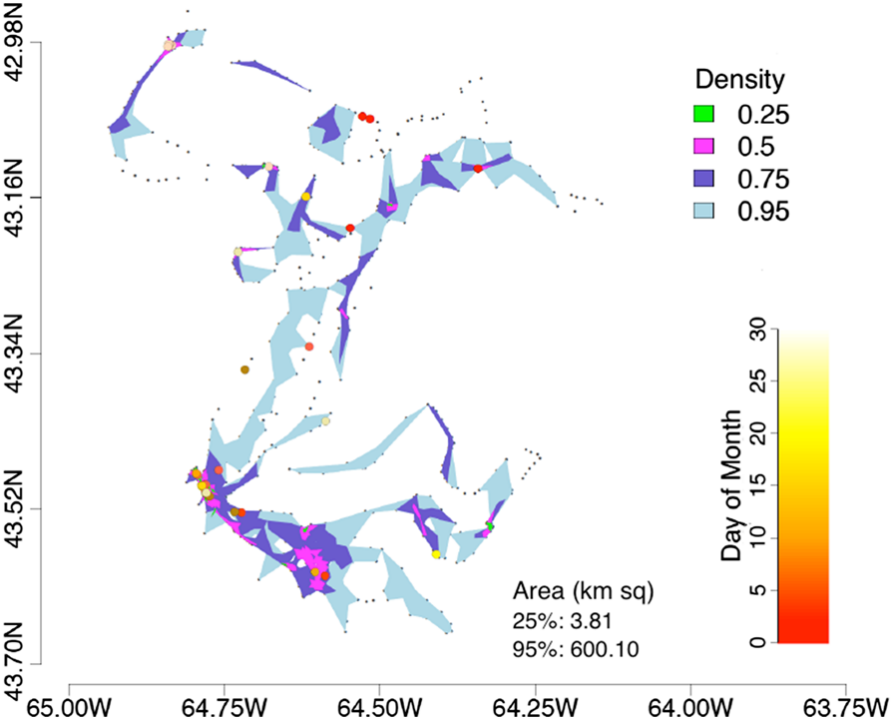
Individual sampling effort. Intensity of area use is represented by density quantiles. Each density quantile contains a percentage of GPS surface locations (25, 50, 75, 95) and is color coded based on intensity of use (bright green=highest, light blue=lowest). Small grey points represent GPS surface locations. Larger, colored points represent 69 kHz transmissions color coded by day of the month. Area estimates (km ^2^) for the 25 % and 95 % density quantiles are shown in the lower right hand corner

Transmissions were received from a broad geographic distribution, though very few were received outside the 75 % density quantile (n =7) (Table 1, Fig. 2). A large cluster of transmissions was received over the course of the month at one location (64.80W, 43.50N, Fig. 2). The highest transmission reception per unit sampling effort (TPUE) occurred in the 25 % density quantile (35.37, SE: 8.94), roughly seven times higher than at the 50 % density quantile (5.26, SE: 1.83).

### Collective area use

**Fig. 3:**
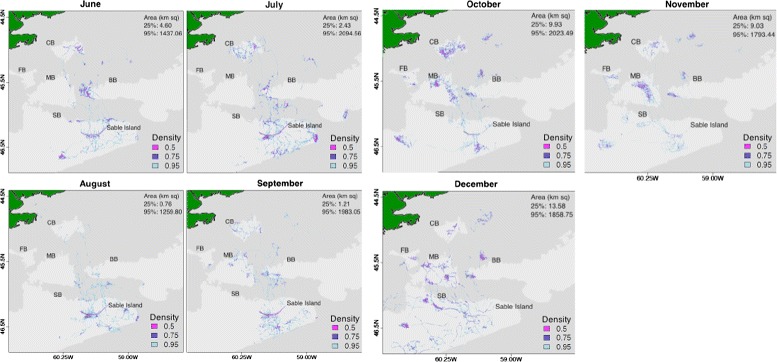
Monthly bioprobe collective area use. Collective area use on the Scotian Shelf by 15 female adult grey seals during the 7-month post-moult pre-breeding period (mid June to December). Banks on the Scotian Shelf are outlined at the 100 m isobaths, and include French (FB), Canso (CB), Middle (MB), Banquereau (BB) and Sable Banks (SB). Intensity of area use is represented by density quantiles containing a percentage of GPS surface locations (50, 75, 95). The 25 % density quantile representing the most intensely used areas was not visible on the current map scale. These areas are located on Sable Island from June-September and over MB, CB, and BB from October-November, and scattered in December. The 95 % density quantile (light blue) corresponds to the overall collective area used (km ^2^). Area estimates (km ^2^) for the 25 % and 95 % density quantiles are shown in the upper right hand corner

The geographic spread in total area sampled and areas intensely used by seals was similar from June through September. In these months, seals spent a large amount of time inshore near Sable Island, which is evident from the high density of locations that outline the island (Fig. 3). Seals tended to make trips immediately south of Sable Island to the edges of Sable Bank (SB), and as far north as Canso Bank (CB), with some foraging east of CB in June, July, and September (Fig. 3). In the autumn and winter months, seals spent increasingly more time-at-sea and less time near Sable Island. Seals began increasingly using French (FB) and Middle Banks (MB) from September-November, with use decreasing slightly in December (Fig. 3). From October to December, seals used areas on the lower part of Banquereau Bank (BB) and immediately above the bank. In October and November, area use occurred in large patches over MB, and over CB in October. In December, seals intensely used small areas to the north and west of Sable Island along SB, with fewer and more directed paths between Sable Island and outlying areas (Fig. 3). These patterns suggest that seals made longer trips and returned less frequently to Sable Island later in the year.

**Fig. 4:**
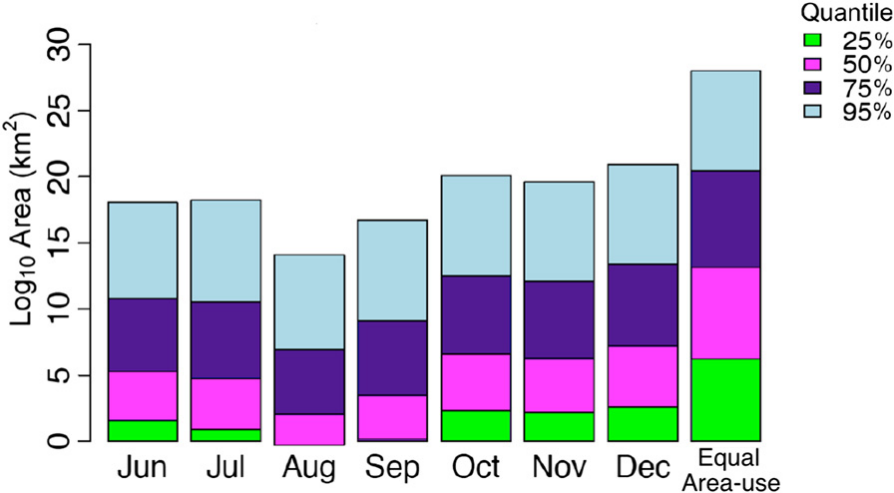
Monthly area use estimates (km ^2^ on log10 scale) for each density quantile (25, 50, 75, 95). Each density quantile contains a percentage of GPS surface locations (25, 50, 75, 95) and is color coded based on intensity of use (bright green=highest, light blue=lowest). The “Equal Area use” bar is the expected area use pattern if all areas are used equally

Seals exhibited more variable patterns of area use during the summer months. Seals covered the smallest area (1437 km ^2^) and spent the least amount of time at sea in June (10,681 GPS locations). Although this could be attributed to the fact that seals were tagged in mid-June (Fig. 3). During this month, surface locations were highly dispersed at both the 25 % and 95 % density quantiles (Fig. 4). Seals spent a large amount of time-at-sea in July (18,335 GPS locations), covering the largest area of the entire study period (2094 km ^2^), which was 1.5 and 1.6 times larger than in the preceding and following months (Fig. 4). While surface locations were highly dispersed at the 95 % density quantile, surface locations were clustered over a small area (2.4 km ^2^) at the 25 % density quantile. In August, seals spent relatively less time at sea (17,065 GPS locations) and more time inshore near Sable Island than in July, making only small trips from Sable Island (Fig. 3). During this time, seals covered the smallest overall area (1259 km ^2^) and intensely used area (0.8 km ^2^), exhibiting the densest clustering of surface locations.

The September-November period was marked by a steady increase in time at sea, spatial extent, and dispersal. At the 25 % quantile of use, spatial extent increased from September (1.2 km ^2^) to October and November (9.9 and 9.0 km ^2^, respectively), reflecting a change from high density to low density of surface locations. In December, seals spent relatively less time at sea (16,611 GPS locations) than in October and November. Surface locations were distributed over a large spatial extent at the 95 % and 25 % density quantiles (1858 km ^2^ and 13 km ^2^ respectively), exhibiting relatively high levels of dispersion.

### Discussion

Our work illustrates the flexibility of the R package, T-LoCoH. It is a tool that can be extended to quantify the total area sampled, and the area intensely sampled by multiple and individual instrumented animals. In comparison to other home-range estimation techniques, LoCoH methods are particularly well suited to the study of collective area use by multiple organisms due to their ability to exclude areas known not be used by the animals [9]. This is achieved by matching the utilization distribution closely to the actual GPS locations. The incorporation of time into the construction of nearest neighbors in the T-LoCoH package provides the user with the ability to extract time- and space-use estimates for the estimated polygons. We demonstrate how these measures of sampling effort can be used to calculate the rate of transmissions received at a location by accounting for differences in the amount of time an individual seal spent in that area. Although we are limited in the inferences we can draw from the transmission receipt rate estimated due to the small sample size of our individual analysis, we believe these data provide a useful demonstration of how this technique may be applied. With a larger sample size, it is easy to envision how estimates of the rate of transmissions received in different areas may be compared to better understand the overlap and perhaps association between these species.

Space-use patterns can change over time in response to environmental variability (e.g., changes in temperature, prey distribution, predator density) and an individual’s age, sex, and life stage [18–21]. When animals are used as bioprobes, these biological processes/responses determine where sampling occurs and have important implications for the inferences we draw. The distribution and intensity of sampling has many implications: first, they determine the area over which we can extend our findings; second, they influence our ability to assess the accuracy of our measurements using repeated samples; third, they determine the biological and physical conditions recorded such as bathymetry, substrate, location, etc.; last, they influence the performance of sampling devices such as acoustic tags [22–27]. Data collected by instrumented animals have greatly contributed to large-scale oceanographic predictive models [28, 29]. In these models, uneven sampling is accounted for in the data assimilation process whereby measurements, such as those collected by bioprobes, are used to adjust the model output and reduce the model uncertainty locally. However, this approach is not suitable for all data types collected by bioprobes. The two extensions of the T-LoCoH package offer key alternative approaches for accounting for changes in sampling effort.

Using T-LoCoH to study collective sampling effort of multiple bioprobes, we found that, in 2011, grey seal bioprobes sampled a small portion of the overall shelf area, with intensely sampled areas heterogeneously distributed in patches separated by large expanses of area used predominantly for travel. In summer and early autumn, sampling occurred over a relatively small portion of the Scotian Shelf, with small patches of heavily sampled areas north and immediately south of Sable Island and the majority of sampling concentrated inshore near Sable Island. In contrast, autumn was marked by increasingly little sampling inshore near Sable Island, with the majority of sampling occurring over a large distribution. During this time, sampling was concentrated in a few large patches, providing solid coverage and repeatable measurements at certain banks. In December, sampling was spread over a much larger expanse of the Scotian Shelf, with intense sampling occurring in many small patches in deeper water at the outer limits of this range, with sampling paths connecting these areas to Sable Island. These patterns are in line with past studies of grey seal area use that found marked seasonal changes in the distribution, diet, and foraging effort according to seasonal changes in their energy needs, and changes in the availability of their prey [12–17]. Breed et al. [16] found that grey seals, especially females, tended to remain inshore near haul-out sites from May through August and make smaller foraging trips (distance and time). Breed et al. [16] also observed increased foraging efforts by grey seals as they near the January breeding period. Grey seals are capital breeders that rely heavily on accumulated energy stores to successfully reproduce [12, 30]. As a result, their movement patterns are expected to change in response to fluctuations in the distribution and availability of their prey [17].

As our knowledge of the processes that drive patterns in animal movement and our ability to predict these patterns in space and time improves, we may be able to use patterns in sampling coverage to further direct sampling in areas where bioprobes are absent using ship-based surveys, gliders, and acoustic arrays. Broad-scale research questions such as the overlap between predators and prey could be addressed by strategic use of other acoustic monitoring devices coincident with the deployment of acoustic transceivers on mobile marine animals.

## Conclusions

Acoustic transceivers show great potential as a tool to study the location and timing of intraspecific interactions such as schooling, spawning aggregations, and mate pair formation, as well as interspecific interactions such as predator-prey and mixed-species aggregations [31]. However, a number of challenges associated with these data remain: first, tag performance varies in response to environmental conditions and must be accounted for [32]; second, we now know that 69kHz acoustic transmissions are within grey seals’ hearing range [33]; lastly, the opportunistic nature of the data violates the assumption that all points in a study area have an equal probability of being sampled [34]. Our manuscript focuses on the final challenge and presents an approach that can be applied to quantify the individual and collective sampling coverage of instrumented grey seals. T-LoCoH is a tool that permits the identification and measure of fine-scale area use that is not possible with kernel smoothing methods. This fine-scale area use when coupled with environmental covariates, should also provide greater insight into the foraging decisions of wide-ranging marine predators and their encounters with potential prey species.

## Methods

### Ethics statement

This research was conducted in accordance with guidelines for the use of animals in research [35] and the Canadian Council on Animal Care. The research protocol for deployment of tags on grey seals was approved by the University Committee on Laboratory Animals, Dalhousie University’s animal ethics committee (animal care protocol: 08-088) and the Department of Fisheries and Oceans (DFO), Canada (animal care permit: 10–65).

### Study site

The study was conducted in 2011 on Sable Island, the Eastern Scotian Shelf, and the southern Gulf of St. Lawrence (Fig. 1). Sable Island is the world’s largest breeding colony for grey seals [36] and the Eastern Scotian Shelf is an important foraging area [4, 15, 37].

### Bioprobe and fish tagging

Twenty female adult grey seals were captured between 11 and 15 June 2011 on Sable Island and each fitted with a VHF transmitter (164–165 MHz, www.atstrack.com), GPS satellite-linked tag (MK10-AF, www.wildlifecomputers.com) and a Vemco Mobile Transceiver (VMT) according to the methods described in Lidgard et al. [4]. In summary, the VHF and GPS tags were attached behind the cranium to maximize the time the GPS tag spent above water in transmission mode. The transceiver was attached to the lower back of the seal to increase the time spent transmitting and receiving detections and to reduce electrical interference with the satellite tag. The GPS tag was programmed to record a location every 15 min. Locations are only recorded when the GPS antenna is above the surface. Although the seal’s diving behavior introduces some irregularity into the timing between location fixes, the intervals are surprisingly “regular” for a diving animal (mean: 17.92, sd: 3.92). GPS attempts were suspended when the unit was dry > 20 min or when a location had successfully been attained. Sixteen instrumented seals were recaptured on Sable Island during the subsequent breeding season (December 2011 to January 2012) and their tags retrieved (median deployment period = 188 d, range = 173–198 d) [38]. In partnership with researchers at the Department of Fisheries and Oceans, Canada (DFO), the Ocean Tracking Network (OTN) tagged a total of 623 Atlantic cod with Vemco V13 acoustic transmitters in the southern Gulf of St. Lawrence (249 between May 2009 and May 2011) and the Eastern Scotian Shelf (374 between November 2010 and November 2012) using methods outlined in Lidgard et al. [11]. During the same period, 298 Atlantic salmon were tagged with V9 or V13 Vemco acoustic transmitters by OTN partners as outlined in Halfyard et al. [39]. Salmon and Atlantic cod tagging locations are shown in Figure two in Lidgard et al. [11].

### Tag data processing

We determined seal locations by analyzing archival GPS data from each tag using software from the manufacturer (Wildlife Computers Data Analysis Programs Version: 3.0.326.0). A location was considered accurate when > 5 satellites were attained, which translated to a residual error < 30 m [40, 41]. Acoustic transmitters and transceivers were programmed to transmit an 8 ping acoustic code every 40–60s or 60–180s, respectively. In order for a tag to be detected, all 8 acoustic pings in the transmission need to be received. Detections of tagged fish recorded by the transceiver are comprised of a date-time stamp and the identities of the transmitting and receiving acoustic tags. The transmission range is estimated to vary between 482.4m in the roughest conditions and 750.4m in the calmest conditions [42]; we predict a more conservative median transmission range of 350–400m for these conditions based on previous analyses of acoustic data in this area [32]. The summarized raw data include all acoustic pings received by the transceiver, including those from incomplete transmissions. We distinguished acoustic pings originating from 69 kHz Vemco transmitters from background noise by the signature intervals between each ping in an acoustic code (Table 2). False detections were identified by VEMCO using proprietary software and removed from the dataset. To link the receipt of partial and complete acoustic transmissions originating from acoustic transmitters attached to fish to locations interpolated at 15 min intervals from the seal’s tracks, clocks in the VMT and GPS tags were synchronized upon deployment. Clocks were time corrected upon retrieval based on the respective clock drift calculated from GPS and VMT tags over the deployment period [4].

**Table 2:** Criteria used to determine ping origins. Adapted from ”Probability of detecting marine predator-prey and species interactions using novel hybrid acoustic transmitter-receiver tags,” by Baker et al., 2014, *PLOS ONE*, 9, e98117

Interval Length	Description
0.26–0.29s	Possible echos or multipath transmissions
0.30–0.70s	Interval range between consecutive pings
0.71–1.50s	Interval range between 1 or more skipped pings
>1.50s	Spurious pings or 3 or more skipped pings

Ping origins deduced from intervals between consecutive pings

### Individual and collective area use

We selected seal 106716 to demonstrate how to calculate the transmission reception per unit effort (Fig. 1a). We sought a seal that had spent little time near other instrumented seals in order to ensure that received transmissions originated from tags deployed on other species. Instrumented seals that remained on the Scotian Shelf were used to study collective area use over this area (Fig. 1b).

### Estimation of area use and intensity

**Fig. 5:**
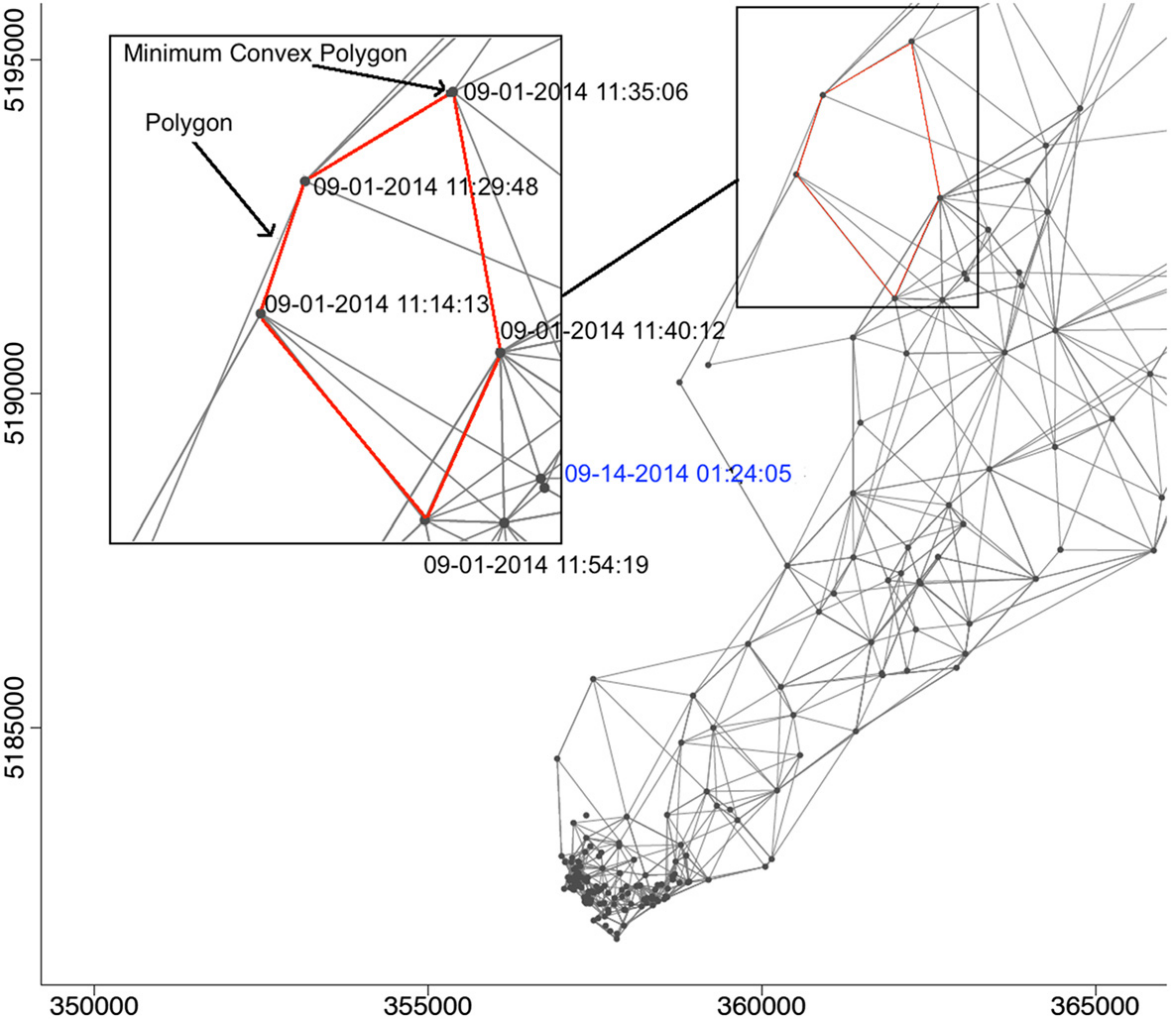
Polygon construction. Polygons (red) are constructed around each GPS location (points) and its nearest neighbours using a time-scaled distance metric *s* that takes into account the time and distance between GPS locations. As a result, GPS locations close in space but far away in time (e.g., blue time-stamp) are not included in the same polygon

We estimated patterns in area use and intensity using the R package, T-LoCoH [43]. T-LoCoH is a non-parametric Lagrangian method for constructing utilization distributions from GPS locations. T-LoCoH expands the base LoCoH algorithm [8] to incorporate the date-time stamp of each location in the selection of nearest neighbours using a time-scaled distance metric (TSD) [10]. The TSD transforms the time interval between any two locations into a third axis of Euclidean space through adaptive scaling of the maximum distance the individual could have travelled during the time interval [10]. Nearest neighbours are therefore determined based on proximity in space and time. We used the *k*-method of sampling to construct polygons around each location and its *k* nearest neighbours (Fig. 5) [10]. This allowed us to standardize the approximate temporal sampling interval of each polygon by including a fixed number of GPS locations. As GPS locations were obtained roughly every 18 min when the seal surfaces (mean: 17.92, sd: 3.92), each polygon was equivalent to approximately the chosen value of *k* multiplied by 18 min.

The T-LoCoH algorithm aggregates local minimum convex polygons (MCPs) constructed around each GPS location to form polygons around each location and its nearest-neighbours (Fig. 5) [10]. Polygons are then sorted based on ascending area. After sorting, polygons are cumulatively merged by taking their union and used to construct density quantiles containing a percentage (25,50,75,95) of locations. We used density quantiles as a measure of intensity of use. The 25 % density quantile represents the most intensely used areas (containing 25 % of locations) and the 95 % density quantile represents the overall area use. Both metrics are in line with traditional home-range methods [8].

### Area use calculation for individual Bioprobes

We used the graphical tools specified in Lyons et al. [10] to select the TSD, *s*=0.03, that resulted in 60 % of polygons being time selected, that is, GPS locations were included or excluded based on time [44]. We selected a nearest-neighbour value of *k*=10, which allowed us to capture the seal’s movement patterns over a 3 h period. We inspected the estimated area by quantile and compared the perimeter:area estimates (edge:area) to ensure that the selected value of *k* did not result in a sudden jump in area [44].

### Area use calculation for multiple Bioprobes

We selected a nearest-neighbour value of *k*=5 to closely fit the GPS locations and standardize polygon temporal range to approximately 90 min. We did not incorporate time into our selection of nearest neighbours as the wide geographic spread of locations at any one time produced spurious results when polygons were constructed using TSD. We were conservative in our choice of *k* because collective area use estimates are more susceptible to the inclusion of unused areas than individual area use estimates for two reasons: (1) time is not included in the selection of nearest neighbours, and (2) GPS locations near one another are not necessarily part of the same animal’s track, making it difficult to know the path trajectory and therefore what areas are used vs. not used. We examined surrounding values of *k* and inspected the estimated area by quantile and perimeter:area curves to ensure that the selected value of *k* did not result in a sudden jump in area [44].

### Spatial and temporal changes in collective area use

We stratified the data by month to compare temporal changes in collective area use. In addition to spatial changes in seal distribution and the distribution of intensely used areas, we compared two metrics of area use at the 95 % and 25 % density quantiles: time-at-sea and area covered (km ^2^). Time-at-sea is equivalent to the number of GPS locations at the 95 % and 25 % density quantiles. GPS locations are taken roughly every 18 min when the animal is in the water, and every 8 days when the animal is hauled-out for >12 hours.

### Relating acoustic data to sampling coverage

We used acoustic pings from 69 kHz transmissions received by seal 106716 in the month of September to demonstrate how to determine the rate of partial acoustic transmissions received irrespective of the time a bioprobe spends in an area. The absence of other instrumented seals in this instance allows for the study of spatial and temporal patterns in encounter rates between this seal and fish species for which there is otherwise no independent location information. We focused on the month of September for two main reasons: first, during this month a high number of 69 kHz acoustic transmissions were received, yielding a reasonable sample size (n=40); second, the seal was isolated from other instrumented seals for the entire month, simplifying the interpretation of the received acoustic transmissions. We chose to analyze 69 kHz acoustic transmissions at the level of individual acoustic pings rather than at the level of the detection (receipt of all 8 individual pings), because numerous environmental conditions [22–27] make it difficult to receive a full transmission. Acoustic pings originating from animals instrumented with Vemco 69kHz acoustic transmitters can be distinguished from pings originating from other sources by the time interval between consecutive pings [32]. While this information alone is not enough to identify the transmitting tag, we can use it to determine important information about where animals tagged with acoustic transmitters are present and absent.

Acoustic pings originating from 69kHz transmissions were matched to the closest GPS location in time recorded for that instrumented seal using the synchronized clocks of the transceiver and GPS transmitter. Using the Intersect tool in ArcMap [45], each GPS location was matched to the polygon in which it was contained. The polygon data contained information on: the polygon reference number, the density quantile the polygon belonged to, the area of the density quantile (km ^2^), the timespan (min) the polygon was occupied for, and the polygon area (km ^2^). We summarized these results for the 25 %, 50 %, and 75 % density quantiles, which had sample sizes of 4 transmissions received or more. The rate of partial transmissions received per unit sampling effort (TPUE) was calculated by dividing the number of transmissions received in a polygon by the sampling effort. Sampling effort was defined as the area of the polygon divided by the time the polygon was occupied for: $\frac {\text {Polygon Area} (km^{2})}{\text {Time (h)}}$.

## References

[1] Hooker SK, Boyd IL (2003). Salinity sensors on seals: use of marine predators to carry CTD data loggers. Deep Sea Research Part I: Oceanographic Research Papers.

[2] Biuw M, Boehme L, Guinet C, Hindell M, Costa D, Charrassin J-B (2007). Variations in behavior and condition of a Southern Ocean top predator in relation to *in situ* oceanographic conditions. Proc Nat Acad Sci.

[3] Wilson SG, Block BA (2009). Habitat use in Atlantic bluefin tuna *Thunnus thynnus* inferred from diving behavior. Endangered Species Res.

[4] Lidgard DC, Bowen WD, Jonsen ID, Iverson SJ (2012). Animal-borne acoustic transceivers reveal patterns of at-sea associations in an upper-trophic level predator. PLoS ONE.

[5] Austin D, Bowen WD, McMillan JI, Iverson SJ (2006). Linking movement, diving, and habitat to foraging success in a large marine predator. Ecology.

[6] Padman L, Costa DP, Bolmer ST, Goebel ME, Huckstadt LA, Jenkins A, et al. Seals map bathymetry of the Antarctic continental shelf. Geophysical Res Lett. 2010; 37(L21601).

[7] Kie JG, Matthiopoulos J, Fieberg J, Powell RA, Cagnacci F, Mitchell MS (2010). The home-range concept: are traditional estimators still relevant with modern telemetry technology?. Philos Trans R Soc B Biol Sci.

[8] Getz WM, Wilmers CC (2004). A local nearest-neighbor convex-hull construction of home ranges and utilization distributions. Ecography.

[9] Getz WM, Fortmann-Roe S, Cross PC, Lyons AJ, Ryan SJ, Wilmers CC (2007). LoCoH: nonparameteric kernel methods for constructing home ranges and utilization distributions. PLoS ONE.

[10] Lyons AJ, Turner WC, Getz WM (2013). Home range plus: A space-time characterization of movement over real landscapes. Mov Ecol.

[11] Lidgard DC, Bowen WD, Jonsen ID, Iverson SJ (2014). Predator-borne acoustic transceivers and gps tracking reveal spatiotemporal patterns of encounters with acoustically tagged fish in the open ocean. Mar Ecol Prog Ser.

[12] Beck CA, Bowen WD, Iverson SJ (2003). Sex differences in the seasonal patterns of energy storage and expenditure in a phocid seal. J Anim Ecol.

[13] Beck CA, Iverson SJ, Bowen WD, Blanchard W (2007). Sex differences in grey seal diet reflect seasonal variation in foraging behaviour and reproductive expenditure: evidence from quantitative fatty acid signature analysis. J Anim Ecol.

[14] Austin D, Bowen WD, McMillan JI (2004). Intraspecific variation in movement patterns: modeling individual behaviour in a large marine predator. Oikos.

[15] Breed GA, Bowen WD, McMillan JI, Leonard ML (2006). Sexual segregation of seasonal foraging habitats in a non-migratory marine mammal. Proc R Soc B Biol Sci.

[16] Breed GA, Jonsen ID, Myers RA, Bowen WD, Leonard ML (2009). Sex-specific, seasonal foraging tactics of adult grey seals (*Halichoerus grypus*) revealed by state-space analysis. Ecology.

[17] Breed GA, Bowen WD, Leonard ML (2013). Behavioral signature of intraspecific competition and density dependence in colony-breeding marine predators. Ecol Evol.

[18] Aarts G, MacKenzie M, McConnell B, Fedak M, Matthiopoulos J (2008). Estimating space-use and habitat preference from wildlife telemetry data. Ecography.

[19] Wakefield ED, Phillips RA, Trathan PN, Arata J, Gales R, Huin N (2011). Habitat preference, accessibility, and competition limit the global distribution of breeding black-browed albatrosses. Ecol Monogr.

[20] Manly B, McDonald L, Thomas D, McDonald T, Erickson W. Resource selection by animals: statistical analysis and design for field studies. Nordrecht, The Netherlands: Kluwer; 2002.

[21] Breed GA, Bowen WD, Leonard ML (2011). Development of foraging strategies with age in a long-lived marine predator. Mar Ecol Prog Ser.

[22] How JR, de Lestang S (2012). Acoustic tracking: issues affecting design, analysis and interpretation of data from movement studies. Mar Freshwater Res.

[23] Gjelland K, Hedger R (2013). Environmental influence on transmitter detection probability in biotelemetry: Developing a general model of acoustic transmission. Methods Ecol Evol.

[24] Finstad B, Økland F, Thorstad EB, Bjørn PA, McKinley RS (2005). Migration of hatchery-reared Atlantic salmon and wild anadromous brown trout post-smolts in a Norwegian fjord system. J Fish Biol.

[25] Singh L, Downey NJ, Roberts MJ, Webber DM, Smale MJ, Van den Berg MA (2009). Design and calibration of an acoustic telemetry system subject to upwelling events. African J Mar Sci.

[26] Heupel MR, Semmens JM, Hobday AJ (2006). Automated acoustic tracking of aquatic animals: scales, design and deployment of listening station arrays. Mar Freshwater Res.

[27] Kuperman WA, Lynch JF (2004). Shallow-water acoustics. Physics Today.

[28] Fedak M. (2013). The impact of animal platforms on polar ocean observation. Deep Sea Research Part II: Topical Studies in Oceanography.

[29] Roquet F, Wunsch C, Forget G, Heimbach P, Guinet C, Reverdin G (2013). Estimates of the Southern Ocean general circulation improved by animal-borne instruments. Geophysical Res Lett.

[30] Iverson SJ, Bowen WD, Boness DJ, Oftedal OT (1993). The effect of maternal size and milk energy output on pup growth in grey seals (*Halichoerus grypus*). Physiol Zool.

[31] Holland KN, Meyer CG, Dagorn LC (2009). Inter-animal telemetry: results from first deployment of acoustic ‘business card’ tags. Endangered Species Res.

[32] Baker LL, Jonsen ID, Flemming JEM, Lidgard DC, Bowen WD, Iverson SJ (2014). Probability of detecting marine predator-prey and species interactions using novel hybrid acoustic transmitter-receiver tags. PLoS ONE.

[33] Stansbury AL, Götz T, Deecke VB, Janik VM (2015). Grey seals use anthropogenic signals from acoustic tags to locate fish: evidence from a simulated foraging task. Proc R Soc B Biol Sci.

[34] Borchers DL, Buckland ST, Goedhart PW, Clarke ED, Hedley SL (1998). Horvitz-thompson estimators for double-platform line transect surveys. Biometrics.

[35] Anon. (2006). Guidelines for the treatment of animals in behavioural research and teaching. Animal Behaviour.

[36] Bowen WD, McMillan J, Mohn R (2003). Sustained exponential population growth of grey seals at Sable Island, Nova Scotia. ICES J Mar Sci Journal du Conseil.

[37] Bowen WD, Beck CA, Iverson SJ, Austin D, McMillan JI (2006). Linking predator foraging behaviour and diet with variability in continental shelf ecosystems: Grey seals of eastern Canada. Conserv Biol Series-Cambridge-.

[38] Baker LL, Flemming JEM, Jonsen ID, Lidgard DC, Iverson SJ, Bowen WD. Data from: A novel approach to quantifying the spatiotemporal behavior of instrumented grey seals used to sample the environment. Movebank Data Repository. 2015. doi:10.5441/001/1.910p0c20. 10.1186/s40462-015-0047-4 PMC4514985 26213626

[39] Halfyard EA, Gibson AJF, Stokesbury MJ, Ruzzante DE, Whoriskey FG, Zech JM (2013). Correlates of estuarine survival of atlantic salmon postsmolts from the Southern Upland, Nova Scotia, Canada. Can J Fisheries Aquat Sci.

[40] Bryant E. 2D location accuracy statistics for Fastloc ®;cores running firmware versions 2.2 & 2.3. Wildtrack Telemetry Systems Ltd. 2007.

[41] Hazel J (2009). Evaluation of fast-acquisition GPS in stationary tests and fine-scale tracking of green turtles. J Exp Mar Biol Ecol.

[42] 69 kHz Sea Water Range Calculator. http://vemco.com/range-calculator. Accessed: 2012-06-04.

[43] Lyons A, Getz W. R Development Core Team: T-LoCoH: Time Local Convex Hull Homerange and Time Use Analysis. 2014. R package version 1.16.

[44] Lyons A. T-LoCoH for R: Tutorial and Users Guide. 2013. http://tlocoh.r-forge.r-project.org.

[45] ESRI (2011). ArcGIS Desktop: Release 10.

